# The Therapeutics of Wakefulness

**Published:** 1869-02

**Authors:** 


The Therapeutics of Wakefulness.—Prof. William A. Hammond, M. D., communicates the following article on wakefulness to the Detroit *Review of Medicine and Pharmacy :*

Brushing the hair, or friction of the skin, as by rubbing the palms of the hands or the backs of the arms, will in some persons tend to induce sleep. Soothing sounds have sometimes a similar effect. On the other hand, persons whose occupations are noisy are apt to awake when the noise to which they are accustomed suddenly ceases. A miller has been known to wake up when the noise of the machinery stopped, and a man who had for many years lived within sound of the roaring of Niagara Falls, was unable to sleep at first on removing from the locality.

But agents more efficacious than such external ones, are those which lessen the amount of blood circulating in the brain. First may be mentioned food and drink, of whose happy influence a frequent illustration is given in the case of a late supper. During digestion more blood circulates through the gastro-intestinal vessels than when the abdominal organs are unemployed ; and this additional amount of blood must come from some other part of the body, since a marked excess of this fluid can not exist in two different parts at the same time, except in case of disease. That the amount of blood in the brain is diminished during digestion is evinced by the feeling of drowsiness commonly experienced, which is a perfectly healthy sensation. The food, thus taken as a therapeutic agent, should be easily digestible. The sensible physician will hardly resort to drugs, if such pleasant medicine as a good supper can be given with equally good effect.

In persons weak or anemic, especially women who have been rendered so by hemorrhages, a dose of some one of the various preparations of alcohol at bed time is frequently advisable. Of these, wines are not generally so admissible as the stronger preparations, such as spirits ; in this country whisky will be most easily attainable. A Methodist clergyman, who came under my care, had been unable, for seven or eight weeks, to sleep more than two hours each night. I prescribed a dose of whisky to be taken at bed time. He at first strongly protested against taking it, upon grounds of principle and his previous habits of total abstinence, but finally agreed to try the remedy. The first night he slept five or six hours, the second seven or eight hours ; his whisky was then reduced in amount gradually, from half a glassful to none at all. He continued to sleep well, and had not formed any habit of drinking.

In healthy persons coffee is calculated to produce wakefulness; in others it acts as a hypnotic, much as other stimulants do in asthenic cases. For the latter purpose, do not trifle with it by administering a little of a weak infusion, but give strong doses at once. Much depends upon the method of making it. Exhaust the strength of three or four ounces of ground coffee by percolation, with rather a small amount of boiling water; and give without milk or cream. Tea is not to be compared with coffee as a therapeutic agent, in this connection. It acts in a similar manner, but not so efficiently.

Sometimes sleep may be produced by physical exercise taken regularly about two hours before bed time. This acts best in asthenic cases It has been often noticed that change of air and carriage exercise produce sleep. The *modus operandi* of this I can not explain, any more than the familiar fact that the rocking of a cradle puts an infant to sleep.

Some time ago, in England, there was constructed a table, known as Darwin's table, for the purpose of producing sleep in the insane. It was circular, and rotated upon a screw at the center. On this the patient was placed, with his head at the center, and the table was turned, thus producing sleep according to correct physiological principles, although these principles were not then known.

The warm bath may be used as a hypnotic. In employing it, the head should be prevented from becoming heated, as by putting cold water upon it while the body is immersed; the application of cold water is, however, rarely necessary in the case of infants. The temperature of the bath is best regulated by the hand. Sometimes cold water alone applied to the head proves sufficient, without the warm bath. I remember having read somewhere in Graves' writings that the Indian women sometimes put their babies to sleep by giving their heads a cold douche; this was also applied in the British army at one time as a punishment, and, it was found, with the almost invariable effect of producing sleep.

Another remedy, often of much value, is the application of a sinapism to the epigastrium. How it acts I do not know; it can not well do so through the circulatory system, but may by impression through the nervous system. The position of the body is important. In many cases, holding the head down produces wakefulness; such persons should, in case of wakefulness, go to sleep in the erect position.

Certain drugs form another class of agents for the production of sleep That which has been longest in use is opium. As regards its power of bringing on sleep, the dose of opium varies in different patients. In small doses of half a grain to three-fourths, as an average, it acts as a stimulant; in moderate doses of one or two grains it is hypnotic; and in larger ones it produces stupor, and not true sleep. Narceine, one of its constituents has been found to produce profound and continuous sleep, but the ordinary preparations of it are too uncertain to be relied upon, and it is too expensive for frequent use.

Hyoscyamus sometimes acts excellently; it has the advantage over opium of not producing headache and constipation the following day. The tincture, especially Neergaard's, may be given in doses of a drachm to a drachm and a half three times a day, if necessary.

Oxide of zinc may prove serviceable in some cases. It came into use in the treatment of the nervous condition preceding delirium tremens. It has also been of value in hysteria when everything else had failed. Its dose is, as a maximum, two grains three times a day; as much as four grains may be given at the same intervals, but this quantity will generally produce irritability of the stomach.

Phosphorus is a remedy which has come into use more recently, in the class of cases of which we are speaking. It is supposed to act by supplying a deficiency in the elements of nervous tissue, increasing the amount of protagon. Owing to its chemical properties, it is not easily administered. It can be given in the form of phosphorated olive oil, in the proportion of four grains to the ounce. It is preferable, however, to boil twelve grains of phosphorus in one ounce of almond oil, and filter. The oil absorbs four grains of phosphorus, so that each minim contains $\frac{1}{120}$ of a grain. Half an ounce of the oil is now mixed with an ounce of gum arabic, and fifteen drops of some aromatic oil are added. Of this mixture the dose is fifteen drops, equal to five drops of the phosphorated oil, and containing $\frac{1}{24}$ of a grain of phosphorus.

I have used this remedy in eight cases with success, and failed in two cases. I try to get three doses taken before bed time, and thus far have generally succeeded in producing the desired effect on the second day, if I had not on the first. The dose may be increased a drop a day, till twenty drops are taken, or signs of gastric irritation supervene. I would not advise giving it in larger doses. In one of my cases, nausea was produced on reaching twenty drops, but sleep ensued also.

But of all the sleep producing agents at our disposal, the bromide of potassium is most deserving the name of hypnotic. I have never seen it fail when given in sufficient quantity. A healthy adult may take from twenty to thirty grains three times a day; the latter dose is not too large when it is needed at all. Sometimes it produces, among its other effects, great weakness in the legs, and a staggering gait, strongly resembling that of a person intoxicated with alcohol. In fact, I know of a gentleman who, while under the influence of this drug, was twice arrested in our streets for drunkenness. Bromide of potassium occasionally produces also a great lowness of spirits, and a disposition to cry. It should be administered very much diluted. It may be conveniently prescribed in one ounce to four ounces of water; a drachm dose of this to be given in at least half a tumblerful of water.

A remedy which I have used recently, especially in cases of nervous excitement where a sedative seemed indicated, is sumbul. This is a plant of the same family as valerian. I have used it in conjunction with bromide of potassium in epilepsy, with the result, as I think, of increasing the effect of the latter. The dose of the fluid extract (Neergaard's) is from twenty drops to a drachm three times a day.

